# Rubella natural immunity among adolescent girls in Tanzania: the need to vaccinate child bearing aged women

**DOI:** 10.1186/s12905-017-0505-9

**Published:** 2018-01-03

**Authors:** Mariam M. Mirambo, Mtebe Majigo, Seth D. Scana, Martha F. Mushi, Said Aboud, Uwe Groß, Benson R. Kidenya, Stephen E. Mshana

**Affiliations:** 1 Department of Microbiology and Immunology, Weill Bugando School of medicine, Catholic University of Health and Allied Sciences, P.O. Box 1464, Mwanza, Tanzania; 2 Department of Microbiology and Immunology, Muhimbili university of Health and allied sciences, P.O. Box 65001, Dar es Salaam, Tanzania; 3 Institute of Medical Microbiology, University Medical Centre Göttingen, Kreuzbergring 57, 37075 Göttingen, Germany; 4 Department of Epidemiology and Biostatics, School of Public Health, Catholic University of Health and Allied sciences, P.O.Box 1464, Mwanza, Tanzania

**Keywords:** Rubella, Adolescent girls, Tanzania, Natural immunity

## Abstract

**Background:**

Rubella primary infection during early stages of pregnancy is associated with high risk of congenital Rubella syndrome (CRS). Prevention of CRS in the resource-limited countries requires multiple strategies. Here, we document the data on the magnitude of Rubella natural immunity among adolescent girls which is a crucial group in devising effective control strategies to prevent CRS.

**Methods:**

A cross sectional study involving 397 adolescent girls was conducted in the city of Mwanza involving five secondary schools. Socio-demographic and other relevant information were collected using pre-tested data collection tool. Rubella IgG antibodies were determined using enzyme immunoassay. The presence of Rubella IgG titers of >10 IU/ml indicated natural immunity.

**Results:**

The mean age of the study participants was 15.18 ± 1.48 years. Of 397 girls, 340 (85.6%) and 57 (14.4%) were from secondary schools representing peri-urban and rural areas, respectively. Out of 397 girls, 90.4% (95% CI: 87-93) were found to be naturally immune with median Rubella IgG antibodies titers of 56.7 IU/ml interquartile range (IQR): 40.8-137. The median Rubella IgG antibodies titers were significantly high in adolescent girls from families with high socio-economic status (63.96 vs. 47.13 IU/ml, *P* < 0.001) and in adolescent girls from peri-urban areas of the city (63.33 vs. 39.9 IU/ml, P < 0.001).

**Conclusion:**

The majority of adolescent girls in the city of Mwanza are naturally immune to Rubella virus. There is a need to compare the effectiveness of screening and vaccinating susceptible adolescent girls with the effectiveness of vaccinating all women of childbearing in controlling CRS in low-income countries.

## Background

Rubella virus infection and congenital Rubella syndrome (CRS) remain to be under recognized public health problems in many low-income countries including Tanzania. In the most of low and middle income countries the introduction of Rubella containing vaccine (RCV) is still not implemented [1]. However, RCV has been widely used in high-income countries with substantial reduction of CRS [2–6]. The ultimate goal for Rubella vaccination is to reduce cases of CRS by ensuring that all women of child bearing age have protective immunity to Rubella virus.

In Tanzania, the median age of first childbirth is about 19 years [7] and about 28% of women are mothers at the age of 18 years [8]. These young women are at increased risk of contracting acute Rubella virus infections as reported by Mwambe et al., [9] which observed that 72% of women who were susceptible to Rubella virus infections were aged between 15 and 24 years. It should be noted that about 90% of infants usually present with CRS if infection occurs in the first trimester [10]. Teratogenic effects and clinical manifestations of the Rubella virus have been found to decrease with gestation age at the time of vertical transmission [11].

In Africa, Rubella natural immunity in adolescent girls has been found to range from 55% to 80% [12–15]. Despite the high seropositivity of Rubella virus specific antibodies among adolescent girls, a substantial number of these girls reach child bearing age without acquiring natural immunity. There is no specific level of natural immunity which is required before routine introduction of RCV. The World Health Organization (WHO) recommends that wide age range campaigns should be conducted as part of RCV introduction. In addition for the effective CRS control the coverage of RCV should be more than 80% to reduce transmission to pregnant women [1] hence decreasing the chances of transmitting the virus to the foetus [16, 17]. According to WHO position paper [1] and Centre for diseases control and Prevention (CDC) [18], different factors have been found to be associated with Rubella immunity. A single dose of RCV in most cases result in long lasting immunity. However, in USA other evidence for Rubella immunity includes receiving more than one dose of RCV and laboratory evidence of >10 IU/ml of Rubella specific IgG antibodies [18]. Other factors such as age, residing in rural areas and socio-economic status(SES) have not been well studied in relation to Rubella immunity but have been found to be associated with immunity in discrete studies [9, 13].

While there are only few studies [9, 19, 20] in Tanzania that have documented the magnitude of the Rubella virus infection among pregnant women, there is no data on the level of natural immunity among adolescent girls who are the main targeted population in effective prevention of CRS. Results of the current study will provide pre-vaccination data that will be important in devising effective vaccination strategies in controlling CRS.

## Methods

This study was conducted in the city of Mwanza between September and October 2014 before the national Rubella vaccination campaigns and at the time of the study no routine Rubella vaccination was provided in both public and private health facilities. Using the prevalence of 89.4% of the population aged between 15 and 24 from Mwambe et al., [9] a minimum sample size obtained using Kish Leslie formula was 145 girls. Considering clustering effect [21] we multiplied the sample size by 1.5 therefore the minimum sample required was 217. However, we enrolled a total of 397 adolescent girls.

From peri-urban areas, out of 20 secondary schools, four schools were randomly selected while out of five schools re-presenting rural areas, one was randomly selected. A total of 940 girls were present in the selected schools. The distance between peri-urban and rural areas is about 20 km. To avoid clustering of the outcome in one category/school, from each school, girls were enrolled randomly to obtain the desired sample size based on the proportion of girls in each school. All girls selected were asked to sign the consent, for those below 18 years they were asked to take the consent forms to the guardian/parents. All guardians/parents consulted agreed participation after more explanations were given by the principal investigator (PI) through telephone. A pre-tested structured data collection tool was used to collect socio-demographic and other general characteristics. After obtaining the written informed consents from participants /parents /guardians, venous blood was obtained in plain vacutainer tubes (Becton Dickinson, UK). Blood was separated to obtain sera and all sera were kept at -80 °C freezer until analysis which was done within 1 month.

Samples were screened for anti-Rubella-specific IgG antibodies using a commercial indirect enzyme linked immunosorbent assay (ELISA® Request-Awareness Technology). All assays were performed according to manufacturer’s instructions. Standard curve was obtained by calibrating the provided standards using ChemWell 2910 Automated EIA (Awareness Technology, Inc.1935 S.W. Martin Hwy. Palm City, FL 34990, and USA). The adolescent girl with anti-Rubella IgG antibody titres of >10 IU/ml was considered naturally immune.

### Data analysis

The data were entered in the computer using excel program and analysed using STATA version 11 (STATA Corp LP, USA). Categorical variables (SES, residence, region etc.) were presented as proportions while continuous variables (age and anti-Rubella IgG titres) were summarized as means and median, respectively. Socio-economic status was defined using mother/father’s education or employment status of parents or sustainable business of the family. Before general analysis, sub-analysis was done for each factor in respect to age category to control clustering of the outcome in one variable. Education level was deleted in the multivariate analysis due to its collinearity effects with age (i.e. clustering those with high classes in older age group). We did univariate and multivariate logistic regression analyses using Random-effects logistic regression model using school as clusters to determine the independent predictors of Rubella natural immunity among adolescent girls. All factors with a *p* value of <0.2 in univariate analysis were subjected to multivariate random-effect logistic regression analysis. Two-sample Wilcoxon rank-sum (Mann-Whitney) test was used to compare median titres for different groups. A p value of <0.05 was considered statistically significant. The odds ratio (OR) and 95% confidence interval (CI) were recorded to describe the strength of associations.

## Results

### Socio-demographic and other characteristics of the study participants

A total of 397 adolescent girls from five secondary schools were enrolled in the study. The mean age of these girls was 15.18 ± 1.48 years. The participation by age was 11(2.8%), 43 (10.8%), 89(22.4%), 73(18.4%), 99(24.9%), 62(15.6%) and 20(5%) for 12, 13, 14, 15, 16, 17 and 18 years, respectively. Of 397 adolescent girls, 302(76.1%) were native of Mwanza region while 95(23.9%) originated from other regions of Tanzania. A total of 300(75.5%) adolescent girls came from families with high SES. As defined by the city council, 340 (85.6%) were from peri-urban and 57(14.4%) were from rural areas (Table 1).

**Table 1:** Univariate and multivariate logistic regression analyses of factors associated with IgG seropositivity among adolescent girls

Characteristics	IgG positivity (N, %)	Univariate		Multivariate	
		OR(95% CI)	*P* value	OR(95% CI)	*P* value
^a^Age(years)	14.97 ± 1.43	1.22(0.95-1.57)	0.116	1.19(0.92-1.50)	0.183
Region					
Mwanza(302)	272(90.1%)	1			
Other (95)	87(91.6%)	1.19(0.28-1.64)	0.399		
Socioeconomic status					
Low (97)	80(82.5%)	1			
High (300)	279(93%)	2.48(0.92-6.70)	0.074	2.29(0.83-6.28)	0.107
Residence					
Rural: (57)	45(78.9%)	1			
Peri-urban: (340)	314(92.4%)	2.27(0.31-16.5)	0.415		
Education level					
Pre form1(43)	38(88.4%)	1			
Form1(144)	130(90.3%)	1.14(0.07-1.7)	0.220		
Form2(63)	52(82.5%)	0.96(0.06-2.34)	0.299		
Form3(147)	139(94.5%)	1.50(0.12-3.3)	0.617		

^a^mean (standard deviation)

### Predictors of rubella natural immunity

**Fig. 1:**
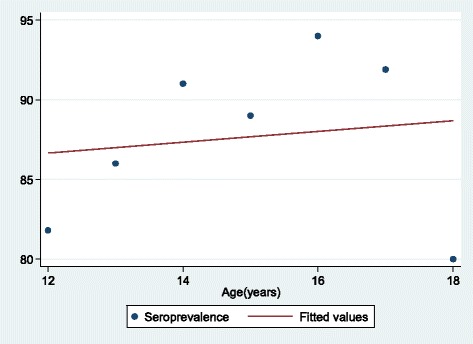
Fitted scattered diagram of seroprevalence vs. age

Of 397 adolescent girls, 359 (90.4%, 95% CI; 87-93) were found to be naturally immune with anti-Rubella IgG titers of >10 IU/ml. On random effect logistic univariate analysis, Rubella natural immunity was found to increase with age but the difference was not statistically significant (OR: 1.22(0.95-1.57, *P* = 0.116). A year increase in age the seropositivity of rubella increases by 0.3% (Fig. 1). It was further observed that no significant difference in Rubella natural immunity was observed between adolescent girls aged <15 years and those aged >15 years (91.2% vs. 88.0%, *p* = 0.302).

On univariate analysis, out of 300 adolescent girls with high SES, 279(93%) were naturally immune compared to 80(82.5%) of those from low SES families (*p* = 0.002). However, when random effect logistic regression analysis was done the association was not significant (OR 2.48, 95% CI; 0.92-6.70, *p* = 0.074). Furthermore, of 340 adolescent girls from peri-urban areas, 314(92%) were naturally immune compared to 78.9% of those from rural areas (*p* = 0.001) (Table 1). As in SES the association was not significant on the random effect logistic regression analysis (OR 2.27, 95% CI; 0.31-16.5, *p* = 0.415).

None of the factor was found to independently predict rubella natural immunity on multivariate random effect logistic regression analysis (Table 1).

### Rubella specific IgG titers

There was no significant difference in median Rubella IgG titers among adolescent girls older than 15 years compared to those younger than 15 years (57.2 IQR 40.78-200 vs. 55 IQR 39.89-95.16, *p* = 0.108). On the other hand, adolescent girls from families with high SES, those originated from other regions and those from peri-urban had significantly higher median tires than counterpart groups as shown in Table 2.

**Table 2:** IgG median titers and its associated factors among adolescent girls with rubella natural immunity

Variables	Median anti-rubella IgG titers (IU/ml)	IQR	*P* value
Age (years)			
> 15(167)	57.21	40.78-200.67	
< 15(192)	55.55	39.89-95.16	0.108
Region(Native)			
Mwanza(272)	52.55	38.57-94.12	
Other(87)	84.24	51.09-205.24	<0.001
Socioeconomic status			
High (279)	63.96	42.95-172.00	
Low (80)	47.13	37.90-58.00	<0.001
Residence			
Peri-urban (314)	63.33	43.17-177.33	
Rural (45)	39.79	34.35-49.09	<0.001

## Discussion

Assessing the Rubella susceptibility profile of the population and the burden of CRS is crucial before the vaccination programme is introduced [22]. Likewise, in the countries where Rubella vaccination programs are introduced surveillance for Rubella outbreaks is emphasized [23]. This is the first study in Tanzania to assess the level of Rubella natural immunity among unvaccinated adolescent girls. The most salient finding in this study is the high level of Rubella natural immunity among adolescent girls aged between 12 and 18 years. Our findings are similar to those reported by Yadav et al., which showed high Rubella seropositivity among child bearing aged women [24]. The other important finding in Tanzania perspective is that no significant difference in the median anti-Rubella IgG titers between adolescents aged ≤15 and those >15 years was observed. Though it might not be generalized to the entire population this study underscores the need for a bigger study to provide data that might lead to the re-visit of the cut-off age which was used during Rubella mass vaccination campaign in 2014. The results of this study cannot be generalized to represent adolescent girls in Tanzania due to the fact that it was done in one region among secondary school girls only. Nevertheless, the results can give a preliminary picture of Rubella natural immunity in Tanzania.

With the tendency of becoming pregnant at young age, our findings are of great importance because about one tenth of these girls in our setting might be susceptible to Rubella virus infection hence at risk of transmitting the virus to the fetus which may result into CRS. These findings are consistent with other studies [25–27] which showed high anti-Rubella IgG seropositivity among adolescent girls. Despite the good strategy of vaccinating children up to 18 months, for the effective CRS prevention, Tanzanian Government should consider addition strategy of vaccinating all women of childbearing age [22]. Reduction in CRS cases will save the Tanzanian government a significant amount of money that will be used in lifetime treatment of these cases. It has been observed that the lifetime treatment of CRS can cost between 14,000USD and 200,000USD [28–30].

Adolescent girls from peri-urban had significantly higher median specific anti-Rubella IgG titers than those from rural areas this might be attributed to recurrent exposure due to high population density in peri-urban areas. However, these findings are inconsistent with previous studies [31, 32] which showed high seropositivity among girls from families with low SES and those residing in rural areas. In comparison to Kenya study by Kombich et al., [13] the mean age of pupils were 11.8 years compared to 15.2 years in the current study. In Kenya study, they attributed low SES and overcrowding. This may perhaps be opposite in Tanzania whereby the majority of the population with high SES live in peri-urban areas where there is overcrowding which consequently favours Rubella virus transmission. The observation is further supported by the fact that the majority of peri-urban girls in the current study are Rubella naturally immune. These findings might be affected by lack of stringent criteria in categorizing the residence whether is peri-urban or rural areas due to lack of clear boundaries. In addition, classification of SES in developing countries is a complex issue because the education of parents or occupation might not correlate with standard of living.

## Conclusion

A significant proportion of adolescent girls in Mwanza city have protective Rubella antibodies with only one tenth of them being susceptible to acute Rubella virus infections. There is a need to compare the effectiveness of screening and vaccinating susceptible adolescent girls to the effectiveness of vaccinating all women of childbearing age in controlling CRS in developing countries.
